# Prognostic value of three-dimensional echocardiographic right ventricular ejection fraction in patients with pulmonary arterial hypertension

**DOI:** 10.18632/oncotarget.13505

**Published:** 2016-11-22

**Authors:** Mitsushige Murata, Toshimitsu Tsugu, Takashi Kawakami, Masaharu Kataoka, Yugo Minakata, Jin Endo, Hikaru Tsuruta, Yuji Itabashi, Yuichiro Maekawa, Mitsuru Murata, Keiichi Fukuda

**Affiliations:** ^1^ Department of Laboratory Medicine, Keio University, Tokyo, Japan; ^2^ Department of Cardiology, School of Medicine, Keio University, Tokyo, Japan

**Keywords:** right ventricular function, right ventricular ejection fraction, pulmonary arterial hypertension, three-dimensional echocardiography, prognosis

## Abstract

**Background:**

Right ventricular (RV) function is an independent predictor of clinical outcomes in patients with pulmonary arterial hypertension (PAH). However, it remains controversial which RV parameter should be measured as an appropriate index for the treatment of PAH. The aim of this study was to identify the most useful parameter that correlates with hemodynamics and predicts clinical outcomes in PAH.

**Results:**

Most of the clinical and echocardiographic RV parameters were significantly correlated with pulmonary vascular resistance (PVR) as well as mean pulmonary arterial pressure (mPAP). Among these, three dimensional right ventricular ejection fraction (3DRVEF) showed the strongest hemodynamic correlation, followed by 6-minute walk distance. Receiver operating characteristic analysis of association with cardiac events including death, hospitalization, and intervention revealed a greater area under the curve for 3DRVEF than for mPAP (0.78 vs. 0.74). Kaplan-Meier analysis showed that patients with 3DRVEF less than 38% had significantly shorter event-free survival than those with greater than 38% (*P* = 0.0007). Finally, the Cox proportional hazards analysis revealed that 3DRVEF, but not mPAP, was an independent predictor of clinical events in PAH.

**Materials and Methods:**

Eighty-six consecutive patients were enrolled in this study. RV hemodynamic parameters were measured by right heart catheterization (RHC). RV function was assessed using two-dimensional speckle-tracking echocardiography and three-dimensional transthoracic echocardiography (3DTTE) to evaluate RV free wall global strain (RVFS) and RVEF.

**Conclusions:**

RVEF measured by 3DTTE could be a useful parameter for noninvasively assessing RV hemodynamics and predicting the clinical outcomes in PAH patients.

## INTRODUCTION

Pulmonary arterial hypertension (PAH) is a progressive disease characterized by elevated pulmonary vascular resistance (PVR) and resulting in right ventricular (RV) failure and death [1]. Accumulating reports have implicated RV function as an independent predictor of prognosis in PAH [2–8]. The RV sensitivity to afterload is heightened compared to the left ventricle (LV) and increased RV afterload can dramatically reduce stroke volume, thus RV dilation might adapt to maintain cardiac output, leading to a decreased RV ejection fraction (RVEF). Indeed, PAH patients show impaired RV longitudinal systolic function, assessed by RV free wall strain, and RV dyssynchrony [9]. Thus, RV dysfunction could be attributable to many factors including RVEF, RV strain, and RV dyssynchrony, and in PAH, although such dysfunction may be induced mainly by the progressive elevation of PVR, the effects of increased afterload on RV remodeling could be diverse, even in the same degree of PAH, probably due to different levels of contractile reserve and myocardial damage.

RVEF is the most commonly used index of RV contractility, and RVEF measured by cardiac magnetic resonance (CMR) might be useful for risk stratification and clinical management of patients with interstitial lung disease [6]. However, RVEF is highly dependent on loading conditions [10, 11], and thus might not always adequately reflect contractility. In PAH, RVEF may be influenced by several factors including RV contractility, disease severity, and RV size. Regarding the RV contractility, RV strain might reflect the regional wall contraction more accurately and precisely than RVEF, although RV strain would be also affected by RV afterload (severity of PAH). RV contraction is sequential, starting with contraction of the inlet and trabeculated myocardium and ending with contraction of the infundibulum. Thus, there is a slight regional time lag of contraction even under non-disease conditions. However, excessive RV dyssynchrony could potentially reduce cardiac output or increase filling pressure [12]. Thus, it remains controversial which of these RV parameters is the most useful to evaluate the mechanism of dysfunction and predict clinical outcomes in PAH. The aim of this study was therefore to evaluate such mechanisms in patients with PAH by comparing the echocardiographic parameters, assessed by speckle tracking echocardiography and three-dimensional transthoracic echocardiography (3DTTE), with hemodynamic parameters measured by right-sided heart catheterization (RHC).

## RESULTS

The clinical characteristics for all subjects in the study (*n* = 86) are given in Table 1. The majority of patients were female and middle aged, as consistent for PAH. The mPAP ranged from 13 to 68 mmHg (median 31 mmHg). Eight patients had normal mPAP (< 20 mmHg) and eleven had borderline mPAP (20–24 mmHg), resulting in the relatively low mean mPAP of 35 mmHg. The pooled data for hemodynamic and echocardiographic parameters are summarized in Table 2. Hemodynamic parameters indicated an increased mPAP and PVR, while LV function was almost normal. The averaged values for echocardiographic RV parameters were borderline normal according to the ASE guideline [13], although RV fractional area change (RVFAC) and 3DRVEF were lower than the normal limits (Table 3).

**Table 1:** Baseline clinical background for enrolled subjects

Subjects	86
Age, y	50 ± 17
Female, *n* (%)	63 (72)
Height, m	1.59 ± 0.58
Weight, kg	58 ± 12
BSA, m^2^	1.6 ± 0.2
WHO functional class	
I	16
II	41
III	29
Medications at baseline, *n* (%)	
PDE-5I, *n* (%)	60 (70)
ERA, *n* (%)	51 (59)
Prostanoid, *n* (%)	24 (28)
CCB, *n* (%)	9 (10)
VKA, *n* (%)	20 (23)
Uric acid, mg/dL	6.1 ± 2.0
BNP, pg/mL	100 ± 200
6MWD, m	378 ± 102

Unless indicated otherwise, data are given as the mean ± SD.

Abbreviations: WHO, World Health Organization; angioplasty; PDE-5I, Phosphodiesterase-5 inhibitors; ERA, Endothelin receptor antagonist; CCB, Calcium channel blockers; BNP, B-Type natriuretic peptide; 6MWD, 6-min walk distance.

**Table 2:** Hemodynamic and echocardiographic parameters for all subjects

Hemodynamics	
Systolic blood pressure, mmHg	111 ± 17
Diastolic blood pressure, mmHg	64 ± 13
Heart rate, beats/min	72 ± 14
Right atrial pressure, mmHg	5.7 ± 2.7
mPAP, mmHg	34 ± 12
PCWP, mmHg	8.7 ± 3.0
PVR, dyne · sec · cm^−5^	564 ± 375
Cardiac index, L/min per m^2^	2.5 ± 0.6
Echocardiographic	
LV end-diastolic diameter, mm	44 ± 5
LV end-systolic diameter, mm	26 ± 5
LV ejection fraction, %	69 ± 8
E, cm/s	69 ± 19
A, cm/s	69 ± 17
E/A ratio	1.1 ± 0.4
E‘, cm/s	12.3 ± 3.8
E/E' ratio	6.1 ± 2.4
IVC diameter, mm	15 ± 4
Peak TR pressure gradient, mmHg	52 ± 20
Right atrial area, cm^2^	18 ± 5

Abbreviations: mPAP, mean pulmonary arterial pressure; PCWP, pulmonary capillary wedge pressure;

PVR, pulmonary vascular resistance; LV, left ventricular; IVC, inferior vena cava; TR, tricuspid regurgitation.

**Table 3:** Echocardiographic right ventricular parameters for all subjects

RV basal diameter, mm	40.1 ± 7.8
RV mid cavity diameter, mm	31.4 ± 6.7
RV longitudinal diameter, mm	72.5 ± 9.2
RV end-diastolic area index, cm^2^/m^2^	10.1 ± 3.3
RV end-systolic area index, cm^2^/m^2^	15.9 ± 5.7
RV fractional area change, %	31 ± 11
TAPSE, mm	19 ± 4
RV S‘, cm/sec	11.4 ± 3.1
RV index of myocardial performance (tissue Doppler)	0.52 ± 0.24
RV longitudinal strain	
Base septal wall, %	−15.3 ± 5.9
Mid septal wall, %	−17.2 ± 6.4
Apex septal wall, %	−10.7 ± 7.8
Base free wall, %	−21.6 ± 9.6
Mid free wall, %	−21.5 ± 8.1
Apex free wall, %	−18.1 ± 7.9
Global, %	−16.7 ± 4.8
RVFS, %	−19.7 ± 6.4
RV-SD6, ms	80 ± 36
3D	
RV end-diastolic volume index, ml per m^2^	52 ± 17
RV end-systolic volume index, ml per m^2^	30 ± 14
RVEF, %	43 ± 12

Abbreviations: RV; right ventricular; TAPSE, tricuspid annular plane systolic excursion; RVFS, RV free wall global strain; RVEF, right ventricular ejection fraction.

### Correlation of RV function with hemodynamics

RV systolic function, as assessed by several different echocardiographic parameters, was significantly correlated with hemodynamics, except for RAP (Table 4). Compared with conventional RV parameters such as Tricuspid annular plane systolic excursion (TAPSE), RVS’, RV index of myocardial performance (RIMP), and RVFAC, RV strain measured by two-dimensional speckle tracking echocardiography (2DSTE), and RV volumetric parameters measured by 3DTTE showed the highest correlation to hemodynamic parameters, with 3DRVEF the strongest parameter.

**Table 4:** Correlation of echocardiographic RV parameters with hemodynamics

RHC parameter	TAPSE		RV S’		RIMP		RVFAC		RVFS		3DRVEF		RV-SD6	
	r	*P*	r	*P*	r	*P*	r	*P*	r	*P*	r	*P*	r	*P*
Mean PAP, mmHg	−0.24	0.02	−0.20	0.07	0.17	0.1	−0.41	< 0.0001	0.51	< 0.0001	−0.62	< 0.0001	0.46	< 0.0001
PVR, WU	−0.34	0.001	−0.31	0.004	0.25	0.02	−0.39	0.0002	0.60	< 0.0001	−0.61	< 0.0001	0.44	< 0.0001
Mean RAP, mmHg	−0.11	0.3	−0.16	0.1	−0.09	0.4	−0.20	0.06	0.14	0.2	−0.31	0.005	0.16	0.1
CI, L/min per m^2^	0.31	0.003	0.43	< 0.0001	−0.14	0.2	−0.30	0.006	−0.39	0.0004	0.26	0.02	−0.23	0.03

Abbreviations: RV indicates right ventricular; RHC, right heart catheterization; PAP, pulmonary arterial pressure; PVR, pulmonary vascular resistance; RAP, right atrial pressure; CI, cardiac index; TAPSE, tricuspid annular plane systolic excursion; RIMP, right ventricular index of myocardial performance; RVFAC, right ventricular fractional area change; RVFS, right ventricular free wall global strain; 3DRVEF, three-dimensional right ventricular ejection fraction.

### Comparison of baseline characteristics of patients with and without clinical events

The primary end point of pre-specified clinical events occurred in 19 of the 86 patients (16%) as follows: 2 (5.9%) deaths, 9 (9.8%) hospitalizations, 1 pulmonary endoarterectomy (PEA), and 7 balloon pulmonary angioplasty (BPA) for deteriorating right-sided heart failure (Table 5). The group with clinical events were older and showed worse symptoms for RV hemodynamics and function than with the group without events, while LV function was comparable between the groups. RV contraction and RV dyssynchrony were also significantly impaired in the group with events, compared to that without events. Among the echocardiographic RV parameters, 3DRVEF showed the strongest difference between groups.

**Table 5:** Comparison of clinical, echocardiographic, and hemodynamic characteristics of the overall cohort based on clinical events

	No event (*n* = 67)	Event (*n*= 19)	*P* Value
Age, y	48 ± 17	58 ± 16	0.02
Weight, kg	57 ± 10	60 ± 18	NS
Height, cm	158 ± 9	160 ± 11	NS
Female, *n* (%)	52 (78)	12 (63)	NS
WHO	2.0 ± 0.7	2.5 ± 0.6	0.009
6MWD, m	400 ± 85	285 ± 117	0.0005
Hemodynamics			
RAP, mmHg	5.2 ± 2.5	7.5 ± 2.6	0.001
mPAP, mmHg	32 ± 12	42 ± 9	0.0005
Cardiac index, l/min per m^2^	2.6 ± 0.6	2.2 ± 0.5	0.002
PVR, WU	6.2 ± 4.1	10.2 ± 5.3	0.0005
Echocardiography			
LVEF, %	68 ± 8	72 ± 9	NS
E/E’	6.1 ± 2.4	6.2 ± 2.4	NS
RA area index, mm^2^/m^2^	11.2 ± 2.7	13.7 ± 4.3	0.01
RV basal diameter, mm	39.2 ± 7.3	43.1 ± 8.9	NS
RV end-diastolic area index, cm^2^/m^2^	14.3 ± 3.7	15.3 ± 4.9	NS
RV end-systolic area index, cm^2^/m^2^	9.7 ± 3.1	11.5 ± 3.8	0.03
RV fractional area change, %	33 ± 10	24 ± 11	0.003
TAPSE, mm	19 ± 4	17 ± 4	0.04
RV S‘, cm/sec	12.0 ± 2.9	9.4 ± 2.9	0.002
RV index of myocardial performance	0.53 ± 0.27	0.53 ± 0.27	NS
Right ventricular longitudinal strain			
Base septal wall, %	−15.5 ± 6.1	−14.5 ± 5.7	NS
Mid septal wall, %	−17.6 ± 6.6	−15.7 ± 5.9	NS
Apex septal wall, %	−11.7 ± 7.0	−7.2 ± 9.2	0.02
Base free wall, %	−22.7 ± 10.1	−17.6 ± 6.7	0.04
Mid free wall, %	−22.1 ± 7.8	−19.3 ± 9.1	NS
Mid free wall, %	−18.8 ± 6.9	−15.7 ± 10.3	NS
Global, %	−17.6 ± 4.3	−14.0 ± 5.8	0.007
RVFS, %	−20.8 ± 6.3	−16.1 ± 5.9	0.004
RV-SD6, ms	74 ± 30	101 ± 47	0.02
3D			
RV end-diastolic volume index, ml/m^2^	49 ± 16	61 ± 18	0.02
RV end-systolic volume index, ml/m^2^	28 ± 13	41 ± 16	0.0012
RVEF, %	46 ± 12	34 ± 11	0.0008

Unless indicated otherwise, data are given as the mean ± SD.

Abbreviations: WHO, World Health Organization; 6MWD, 6-minute walk distance; RAP, right atrial pressure; mPAP, mean pulmonary arterial pressure; PVR, pulmonary vascular resistance; RA, right atrial; RV, right ventricular; TAPSE, tricuspid annular plane systolic excursion; RVFS, right ventricular free wall global strain; 3DRVEF, three-dimensional right ventricular ejection fraction.

### Association of clinical, echocardiographic, and hemodynamic parameters with clinical events

**Figure 1:**
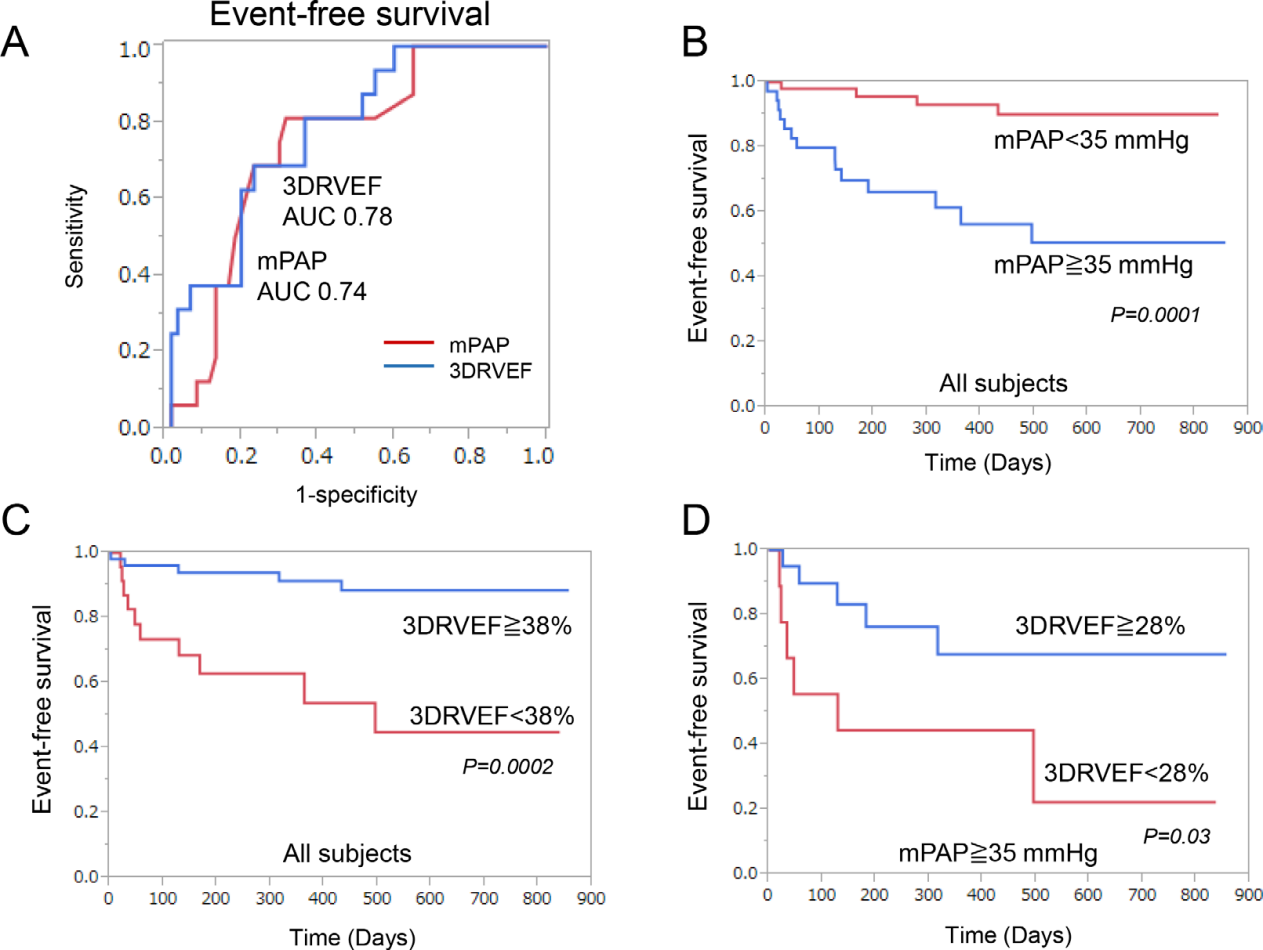
Correlation with clinical events (**A**) Receiver-operating characteristic curve analysis revealed that 3DRVEF was superior to mPAP in predicting associated clinical events. (**B**) Event-free survival of patients with a mPAP ≧ 35 mmHg compared to patients with a mPAP < 35 mmHg in all subjects. (**C**) Event-free survival of patients with a 3DRVEF ≧ 38% compared to patients with a 3DRVEF < 38% in all subjects. (**D**) Event-free survival of patients with a 3DRVEF ≧ 28% compared to patients with a 3DRVEF < 28% in patients with severe PAH (mPAP > 35 mmHg). 3DRVEF = three-dimensional right ventricular ejection fraction, mPAP = mean pulmonary arterial pressure.

The univariate Cox proportional hazards analysis showed that most parameters were significantly associated with clinical events, while some of them including sex, LVEF, RVD, RV end-diastolic area index (RVEDAI), and RIMP had no correlation (Table 6). Multivariate Cox proportional hazards analysis revealed that 3DRVEF and 6MWD, but not mPAP, were independent predictors of clinical events. As shown in Figure 1A, The receiver-operating characteristic curve (ROC) analysis of association with clinical events identified a baseline mPAP of 35 mmHg and a baseline 3DRVEF of 38% as the best cutoff values for predicting clinical events (mPAP: area under the curve 0.76, sensitivity 79%, specificity 69%, *P* value 0.0009, 3DRVEF: area under the curve 0.78, sensitivity 69%, specificity 77%, *P* value 0.0004). Kaplan-Meier analysis demonstrated that long-term outcomes for patients with mPAP ≧ 35 mmHg were worse than for those with mPAP < 35 mmHg (log-rank *P* = 0.0001, Figure 1B). Furthermore, ROC analysis identified a 3DRVEF of 38% as the best cutoff value for predicting clinical events in patients with severe PAH (AUC 0.67, sensitivity 46%, specificity 84%, *P* value 0.06), and Kaplan-Meier analysis revealed that long-term outcomes for patients with 3DRVEF < 38% were worse than for those with 3DRVEF ≧ 38% (log-rank *P* = 0.0002, Figure 1C). Although long-term outcomes for patients with mPAP ≧ 35 mmHg were poor, some of those patients with preserved 3DRVEF (≧ 28%) actually had a better prognosis (Figure 1D).

**Table 6:** Univariate and multivariate cox proportional hazards analysis of the relationships with clinical events

Variable	HR	95% CI	*P* Value
**Univariate**			
Age	1.04	1.01–1.07	0.01
Sex	1.54	0.54–3.96	NS
WHO	2.79	1.35–6.33	0.005
6MWD, m	0.99	0.98–0.99	< 0.0001
logBNP, pg/mL	5.02	2.40–10.6	< 0.0001
Hemodynamics			
Mean PAP, mmHg	1.06	1.03–1.10	0.0009
RAP, mmHg	1.28	1.09–1.49	0.003
PVR, WU	1.15	1.07–1.22	0.0006
Cardiac index, l/min/m^2^	0.18	0.06–0.49	0.0005
Echocardiography			
RA area, cm^2^	1.20	1.06–1.34	0.006
LV ejection fraction, %	1.05	0.99–1.12	NS
RVD, mm	1.70	0.99–2.70	NS
RV end-diastolic area index, cm^2^/m^2^	1.08	0.97–1.19	NS
RV end-systolic area index, cm^2^/m^2^	1.16	1.03–1.29	0.02
RV fractional area change, %	0.94	0.90–0.98	0.002
TAPSE, mm	0.85	0.74–0.98	0.02
RVS’, cm/s	0.82	0.72–0.94	0.004
RV index of myocardial performance	1.21	0.12–4.26	NS
RV end-diastolic volume index, ml/m^2^	1.04	1.01–1.07	0.02
RV end-systolic volume index, ml/m^2^	1.06	1.02–1.09	0.0008
3DRVEF, %	0.92	0.88–0.96	0.0001
RVFS, %	1.12	1.04–1.22	0.003
RV-SD6, ms	1.02	1.01–1.03	0.002
**Multivariate**			
6MWD	0.99	0.98–0.99	0.03
mPAP	0.98	0.90–1.04	NS
3DRVEF	0.91	0.84–0.98	0.01

Abbreviations: CI, confidence interval; HR, hazard ratio; WHO, World Health Organization; 6MWD, 6-minute walk distance; RAP, right atrial pressure; mPAP, mean pulmonary arterial pressure; PVR, pulmonary vascular resistance; RA, right atrial; RV, right ventricular; TAPSE, tricuspid annular plane systolic excursion; 3DRVEF, three-dimensional right ventricular ejection fraction; RVFS, right ventricular free wall global strain.

## DISCUSSION

This study demonstrated that echocardiographic 3DRVEF significantly correlated with invasively measured hemodynamics and thus could be a noninvasive indicator of significant PAH hemodynamics. Furthermore, 3DRVEF was an independent and the strongest predictor of clinical events in patients associated with PAH, implicating the predominant significance of echocardiographic 3DRVEF compared with hemodynamic parameters including mPAP and PVR.

### Comparision of echocardiographic assessment with clinical and hemodynamic conditions

As shown in previous studies, conventional RV parameters including TAPSE [14, 15], RVFAC [2], and RV S’ [14] correlated with cardiac hemodynamics, albeit weakly. However, there are few reports that simultaneously compare the correlations of these parameters. The results presented herein comparatively analyzed both clinical, hemodynamic data and echocardiographic RV function including RV contraction and RV dyssynchrony, and confirmed the prognostic significance of WHO functional class, 6MWD, cardiac index, and most of the echocardiographic RV parameters. Importantly, univariate and multivariate Cox proportional hazard analysis identified echocardiographic 3DRVEF, but not mPAP, as an independent predictor of clinical events (Table 6). These results emphasize the predominant significance of RV function assessed by 3DRVEF, compared with hemodynamic parameters.

### Prognostic value of echocardiographic 3DRVEF in patients with PAH

Previous studies showed that mortality in PAH patients is associated with both the severity of symptoms and the extent of right heart failure, [16] and that RV function is a predictor of mortality in these patients, as evidenced by the correlation between clinical outcomes and several RV parameters including TAPSE, RV S’, RVFAC, RV strain, and RVEF measured by CMR and 3DTTE [17, 5]. Furthermore, Ryo et al. reported that 3DRVEF was a predictive parameter for association with the combined end point of hospitalization, death, or lung surgery [18]. Since RV systolic function is mediated by various factors such as RV contractility, RV synchronization, and PVR [1], the mechanisms for RV dysfunction should be identified for each pathophysiological condition. In the present study, we investigated all the echocardiographic and hemodynamic parameters associated with RV function, and found that among many RV parameters including RV strain, and RV dyssynchrony, 3DRVEF showed the strongest correlation with hemodynamics and prognosis. Historically, hemodynamic parameters including cardiac index and mPAP are used as prognostic predictors in PAH [19], with RV strain and RV dyssynchrony also used to predict clinical outcomes [17, 20]. However, consistent with our results, CMR studies demonstrated the prognostic importance of a decrease in RVEF [8, 21], which also accompanies late disease progression [22]. We postulate that 3DRVEF could be the most useful overall predictive parameter because it reflects both the cardiac systolic and hemodynamic functions associated with prognosis, since RVEF would be affected by RV contraction, RV dyssynchrony, and RV afterload [1, 23]. Further studies are now clearly warranted to clarify the effect of RVEF on clinical outcomes in PAH.

### Clinical implications

Although RHC is necessary for the diagnosis of pulmonary hypertension, repetitive RHC may not be applicable as a routine examination. The present study demonstrated the feasibility and convenience of noninvasive RV analysis using 3DTTE rather than RHC to evaluate the pathology in patients with PAH. The diagnosis of PAH is defined as mPAP > 25 mmHg, which could be reliably detected by 3DRVEF measurments with a cutoff value of 45% (AUC 0.81, sensitivity 60%, specificity 92%, *P* = 0.002) and ePASP with a cutoff value of 36 mmHg (AUC 0.82, sensitivity 64%, specificity 67%, *P* = 0.001).

In the present study, 36 patients had severe PAH (mPAP > 35 mmHg) with a poor prognosis (50% of event-free survival in 2.5 years) (Figure 1D). Notably, among this group, patients with 3DRVEF ≧ 28% had a better prognosis of 70% event-free survival in 2.5 years. Thus, risk stratification by 3DRVEF might identify PAH patients at higher risk of clinical deterioration. This is important, as timely intensification of PAH-specific therapy could prevent further clinical worsening and death.

### Study limitations

This study was a single-center and retrospective cohort study with a small number of patients. Thus, the findings should be prospectively confirmed in a larger population. In addition, subjects in our investigation were chosen as the patients who underwent RHC on suspicion of PAH, implicating that the population included heterogeneous etiology that may weaken or modify the results.

## MATERIALS AND METHODS

The ethics committee of Keio University Hospital approved this study, which comprised a retrospective analysis of patient records from a prospectively maintained registry of patients admitted to the Keio University Hospital. Of 198 consecutive patients who underwent RHC to diagnose pulmonary hypertension from September 2013 to December 2015, 134 patients assessed as eligible (68%) underwent echocardiography including speckle tracking and three-dimensional echocardiography. Finally, 86 patients were included in the main outcome analysis, with 48 excluded due to congenital shunt disease (26), left-sided heart failure (4), or unanalyzable poor trace (18). Among 86 patients, 29 had idiopathic PAH, 6 heritable PAH, 17 collagen diseases, 4 pulmonary diseases, 22 chronic thromboembolic pulmonary hypertension (CTEPH), and 8 other etiologies including blood diseases.

### Right-sided heart catheterization

All patients underwent RHC using a 6- or 7-Fr Swan-Ganz catheter (Swan-Ganz CCO CEDV; Edwards Life Sciences, Irvine, CA, USA). PAP, right atrial pressure (RAP), PCWP, and cardiac output (CO) were measured by RHC. CO was assessed using the thermodilution method or Fick technique, and cardiac index (CI) was calculated by dividing CO by body surface area. PVR was calculated using the follow formula: PVR = (mPAP – mean PCWP)/CO.

### Echocardiographic measurements and analyses

Patients underwent echocardiography using a Vivid-E9 ultrasound system (GE Healthcare, Horten, Norway). Established criteria were used to measure RV size and function [13]. RV end-diastolic area and end-systolic area were assessed using manual planimetry in the RV-focused apical four-chamber view (RV 4CV), and then RVFAC was calculated. TAPSE was measured at the RV 4CV by achieving proper orientation for M-mode measures. RIMP was determined as the sum of the isovolumic contraction time and isovolumic relaxation time divided by ejection time. RAP was estimated based on the diameter of the inferior vena cava (IVC) from the long-axis subcostal view and its phasic response to respiration. RAP was estimated to be 3 mmHg if the IVC was < 21 mm in diameter and collapsed by > 50% at the junction of the hepatic veins with a sniff, or 15 mmHg if the IVC was > 21 mm in diameter and collapsed by < 50% with a sniff. Indeterminate cases in which the IVC diameter and collapse did not fit this paradigm were assigned an intermediate RA *P* value of 8 mmHg. The peak tricuspid regurgitation (TR) velocity (m/s) was determined with continuous-wave Doppler using the highest velocity obtained from multiple views, and the TR pressure gradient (TRPG) was calculated as 4 × (peak TR velocity) (2). RV systolic pressure was obtained by summing TRPG and RAP. The velocity, S′, of RV was obtained by tissue Doppler imaging from RV-focused views at the lateral corner of the tricuspid annulus.

RV global and systolic function was also estimated by 2DSTE. Gray scale imaging of the RV 4CV was obtained with a frame rate of 40–80 Hz, and recordings were processed with acoustic-tracking software (EchoPAC; GE Healthcare), allowing off-line semi-automated speckle-based strain analysis, as described previously [24]. Briefly, lines were first manually traced along the RV endocardium before an additional epicardial line was automatically generated by the software to create a region of interest. After manually adjusting the shape of the region of interest, the software divided the RV region into six segments and generated a longitudinal strain curve. We set the zero strain point as the time from the beginning of the QRS wave on the electrocardiogram, and measured negative peak strain during ventricular systole. In addition, RV dyssynchrony was quantified using the standard deviation of the heart rate-corrected intervals from QRS onset to peak systolic strain for the six segments (RV-SD6), as described previously [16, 25].

Three-dimensional echocardiographic images were obtained from the apical window with the patient in the same position as for 2D echocardiography, as described previously [24]. Briefly, echocardiographic images were stored digitally for offline analysis using TomTec 4D RV Function software (4D analysis; TomTec, Munich, Germany). Within the 3D data set, three orthogonal main cut planes were selected to define the end-diastolic and end-systolic frames within the sequence, as well as several landmarks. On the basis of the initial view adjustment and landmarks, the program automatically provided 4CV, sagittal, and coronal RV views. RV end-diastolic volume, RV end-systolic volume, and RVEF were measured from each 3D echocardiographic data set.

The intra- and inter-observer reproducibility of strain parameters was shown to be acceptable. The intra- and inter-observer variabilities for 3DRVEF were 1.4 ± 3.5% (95% CI: −5.6 to 8.4), and 1.5 ± 3.8% (95% CI: −6.3 to 9.2), respectively.

### Definitions of clinical outcomes

Long-term unfavorable outcome events were pre-specified as primary end points of death, hospitalization, or intervention including PEA or BPA for deteriorating right-sided heart failure. Mean follow-up was 423 days.

### Statistical analysis

Quantitative results are expressed as mean ± SD. Two-group comparisons were performed with unpaired Student *t* tests for means if the data were normally distributed or with Wilcoxon rank-sum tests if the data were not normally distributed. Chi-square or Fisher exact tests were used to analyze the categorical data. Linear regression analysis was used to correlate two parameters. We calculated the cumulative incidence of events using the Kaplan-Meier method and compared the two curves with a log-rank test. We used Cox proportional hazards models to estimate hazard ratio (HR) for cardiovascular events and 95% confidence interval. A multivariate-Cox proportional hazard model was also developed using stepwise regression by selecting those variables that were significant upon univariate analysis. Intra-observer variability and inter-observer variability of 3DTTE and RV longitudinal strain were calculated by a blinded repeat analysis of 20–25 randomly chosen patients. Intra-observer variability was performed 3 months after the first reading to avoid recall bias. Reproducibility was assessed by coefficient of variation. All statistical analyses were performed using JMP 11.0 software (SAS Institute, Cary, NC, USA).

## CONCLUSIONS

RVEF measured by 3D echocardiography could be a useful parameter for noninvasively assessing RV hemodynamics and predicting clinical outcomes in patients with PAH.
